# Synchronous bilateral pheochromocytomas and paraganglioma with novel germline mutation in *MAX*: a case report

**DOI:** 10.1186/s40792-017-0408-x

**Published:** 2017-12-28

**Authors:** Masahiro Shibata, Takahiro Inaishi, Noriyuki Miyajima, Yayoi Adachi, Yuko Takano, Kenichi Nakanishi, Dai Takeuchi, Sumiyo Noda, Yuichi Aita, Kazuhiro Takekoshi, Yasuhiro Kodera, Toyone Kikumori

**Affiliations:** 1 Department of Breast and Endocrine Surgery (Surgery II), Nagoya University Graduate School of Medicine, 65 Tsurumai-cho, Showa-ku, Nagoya, 466-8550 Japan; 2 Faculty of Medicine, Division of Sports Science/Laboratory Medicine, University of Tsukuba, 1-1-1 Tennodai, Tsukuba, Ibaraki 305-8575 Japan; 3 Department of Gastroenterological Surgery (Surgery II), Nagoya University Graduate School of Medicine, 65 Tsurumai-cho, Showa-ku, Nagoya, 466-8550 Japan

**Keywords:** Pheochromocytoma, Paraganglioma, *MAX* mutation

## Abstract

**Background:**

Recent advance of genetic testing has contributed to the diagnosis of hereditary pheochromocytoma and paraganglioma (PPGL). The clinical characteristics of hereditary PPGL are varying among the types of mutational genes. It is still difficult to specify the pathognomonic symptoms in the case of rare genetic mutations. Here, we report the case of synchronous bilateral pheochromocytomas and paraganglioma with novel MYC associated factor X (MAX) gene mutation.

**Case presentation:**

A 24-year-old female had hyperhidrosis and hypertension. Her urine test showed high normetanephrine and vanillylmandelic acid. Enhanced computed tomography revealed three enhanced masses in right adrenal gland, left adrenal gland, and left renal hilus. She was diagnosed with PPGL. Because ^123^I-metaiodobenzylguanidine scintigraphy indicated the accumulations in the left adrenal gland mass and the left renal hilus mass and not in the right adrenal gland mass, we performed laparoscopic left adrenalectomy and extirpation of the left renal hilus mass to preserve the right adrenocortical function. However, her symptoms recurred shortly after the operation presumably due to unveiling of the activity of the right pheochromocytoma. Following right adrenalectomy as the second operation, the catecholamine levels declined to normal range. Her genetic testing indicated the novel germline mutation in *MAX* gene (c.70_73 del AAAC/p.Lys24fs*40).

**Conclusions:**

*MAX* germline mutation is recently identified as a rare cause of hereditary PPGL. The deletion mutation in *MAX* gene in this patient has never reported before. In the case of bilateral pheochromocytomas, the surgical indication should be decided considering each patient’s genetic background. Due to the possibility for other types of malignant tumors, close follow-up is essential for *MAX* mutation carriers.

## Background

Pheochromocytoma and paraganglioma (PPGL) are neuroendocrine tumors that arise from adrenal or extra-adrenal chromaffin cells, and they can occur either sporadically or hereditary [1]. Diagnosing PPGL as hereditary or sporadic is mandatory, because fatal illnesses other than PPGL may occur in some hereditary syndromes. Although multiple endocrine neoplasia type 2, von Hippel-Lindau disease, and neurofibromatosis type 1 have been studied well among hereditary PPGLs, recent advance of genetic analyzing technologies have discovered new causal genes [1]. Currently, about 30% of PPGLs are considered to be caused by germline mutations [2, 3], and their clinical features, such as localization, age on onset, and malignant potential are varying among each patient’s genetic background [1]. However, the characteristics of PPGL with some types of gene mutations have not been fully elucidated due to their rarity.

We experienced a case of synchronous bilateral pheochromocytomas and paraganglioma with novel MYC associated factor X (MAX) gene mutation. There have been few reports of PPGL with *MAX* mutation. We herein report this case and review the related articles.

## Case presentation

A 24-year-old female presented to the regional hospital with complaints of hyperhidrosis and hypertension. She did not have specific past histories or family history of PPGL. Her urine test indicated elevated normetanephrine (NM) and vanillylmandelic acid (VMA) excretion. Then, she was referred to our hospital.

**Fig. 1:**
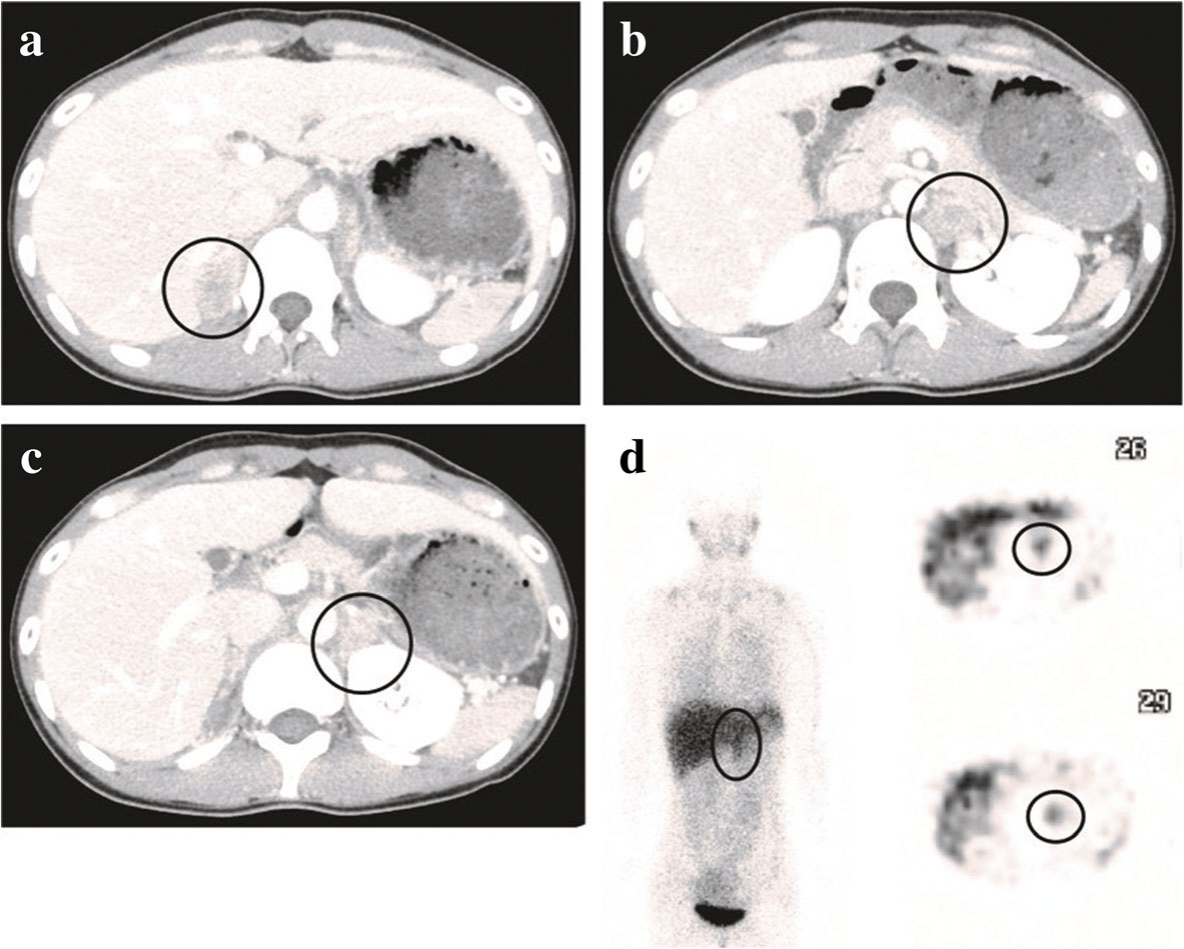
Contrast-enhanced CT showed enhanced masses in the right adrenal gland (**a**), left adrenal gland (**b**), and left renal hilus (**c**). **d** ^123^I-MIBG scintigraphy indicated the accumulations in the left adrenal gland mass and the left renal hilus mass

The patient’s blood pressure was 147/106 mmHg. The results of her 24-h urine collection test are shown in Table 1, which indicated excess production of catecholamine. Contrast-enhanced computed tomography (CT) indicated three enhanced masses in the right adrenal gland (3.5 × 2.2 cm; Fig. 1a), left adrenal gland (2.4 × 1.9 cm; Fig. 1b), and left renal hilus (2.4 × 1.8 cm; Fig. 1c). Abdominal magnetic resonance imaging showed that all three masses were hyperintense in T2 weight image. ^123^I-metaiodobenzylguanidine (MIBG) scintigraphy indicated the accumulations in the left adrenal gland mass and the left renal hilus mass (Fig. 1d). The accumulation was not detected in the right adrenal gland mass. Based on these results, she was diagnosed as bilateral pheochromocytomas and paraganglioma in the left hilus. Because the hormonal activity of the right pheochromocytoma was presumed to be low from the result of MIBG scintigraphy, we planned left adrenalectomy and extirpation of the left hilus mass to preserve right adrenocortical function. She preoperatively took doxazosin up to 8 mg/day for alpha-blockade.

**Table 1:** Results of catecholamine metabolite ratios in 24-h urine collection tests

	Pre-1st operation	Post-1st operation	Pre-2nd operation	Post-2nd operation	Normal range (mg/day)
Methanephrine	0.15	0.09	0.08	< 0.01	0.05–0.20
Normetanephrine	4.10	2.90	3.50	0.27	0.10–0.28
Vanillylmandelic acid	27.60	16.50	22.00	3.40	1.4–4.9

**Fig. 2:**
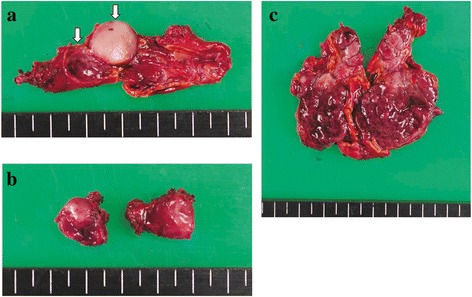
**a** Left adrenal gland resected in the first operation. Multifocal nodules were observed in cross-section (shown by white arrows), and whole adrenal medulla thickened. **b** The cross-section of the left renal hilus tumor resected in the first operation. **c** Right adrenal gland resected in second operation. The adrenal medulla thickened, and the cortex was thin

**Fig. 3:**
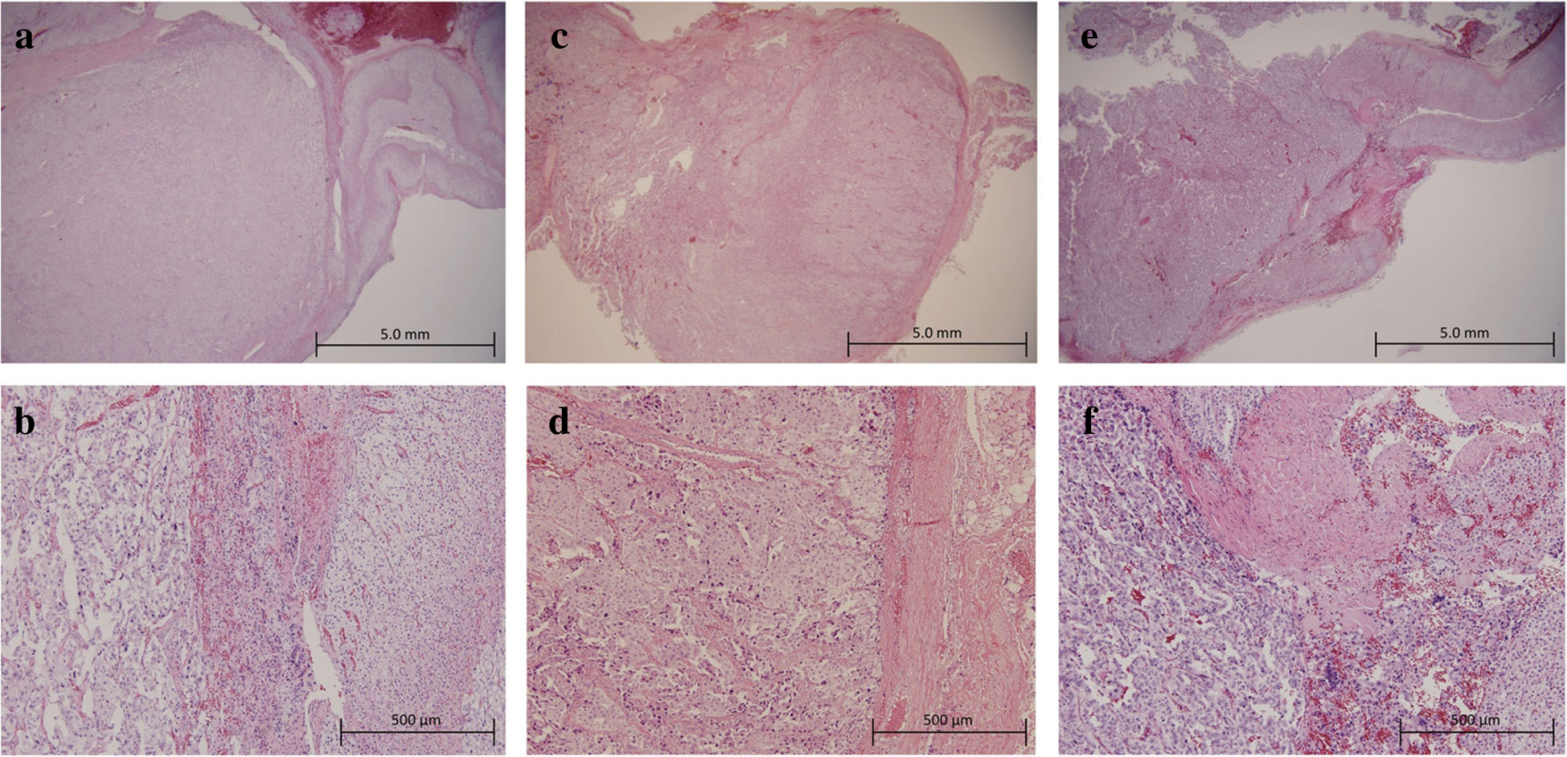
Histopathological results of resected PPGLs (hematoxylin-eosin staining). **a**, **b** Left pheochromocytoma resected in the first operation (**a** low magnification, **b** high magnification). **c**, **d** Paraganglioma in the left hilus resected in the first operation (**c** low magnification, **d** high magnification). **e**, **f** Right pheochromocytoma resected in the second operation (**e** low magnification, **f** high magnification)

The patient underwent laparoscopic left adrenalectomy and extirpation of the left renal hilus mass (operating time: 4:29, blood loss: 62 g). Multifocal nodules and the thickened adrenal medulla were observed in the cross-section of the left adrenal gland, whereas the adrenal cortex was thin (Fig. 2a). The cross-section of the left renal hilus mass is shown in Fig. 2b. The pathological finding indicated that they were PPGL without invasion to surrounded tissues or blood vessels (Fig. 3a–d). After the operation, her blood pressure declined to normal range. On the fourth postoperative day, she complained of hyperhidrosis, and her blood pressure was elevated again. After administration of 4 mg doxazosin, her symptoms ameliorated. Her 24-h urine collection test showed that NM and VMA were still at high levels (Table 1), which suggested that her right pheochromocytoma actively released catecholamine. Because she was highly suspected to have some germline gene mutation associated with PPGL, we performed genetic testing under her and her family’s consent. Genetic testing regarding hereditary PPGL has been approved by the institutional review board in our hospital. PCR-direct sequencing method showed that she had heterozygous germline mutation in *MAX* (c.70_73 del AAAC/p.Lys24fs*40). Accordingly, due to four-nucleotide deletion in exon 3, premature termination of translation is anticipated leaving 62-residue polypeptide, whereas *MAX* gene usually codes 160 amino acids.

Four months after the first operation, laparoscopic right adrenalectomy as the second operation was performed (operating time: 2:26, blood loss: 31 g). The cross-section of the resected right adrenal gland indicated that the adrenal medulla also thickened and the cortex was thin (Fig. 2c). The pathological finding showed pheochromocytoma with capsular and vascular invasion (Fig. 3e, f). In her 24-h urine collection test after the operation, NM and VMA declined to normal range (Table 1). She has not had any evidences of recurrence over a current follow-up period of 2 years. She has not had the symptom of hyperhidrosis, and her blood pressure is normal without any antihypertensive drugs.

### Discussion

MAX is the most conserved dimerization component of the MYC-MAX-MXD1 network, and they work as transcription factors that regulate cell proliferation, differentiation, and apoptosis [4]. Whereas heterodimerization of MAX with MYC acts as transcriptional activators, heterodimers of MAX with MXD1 repress the MYC-dependent transcriptional activities by antagonizing MYC-MAX function [5]. This is why *MAX* is considered as a tumor suppressor gene. *MAX* gene mutations were identified as one of the causes of hereditary PPGLs by the next-generation whole exome sequencing in 2011 [6]. Among 1694 PPGL patients without germline mutations in *RET*, *VHL*, *SDHB*, *SDHC*, *SDHD*, and *TMEM127* genes, 16 heterozygous variants of *MAX* were identified in 23 patients. According to the clinical and biochemical features in 19 PPGL patients with *MAX* germline mutations, seven (37%) patients had a family history of PPGL, and age at diagnosis was relatively young (median age 34 years old). Thirteen (68.4%) patients had bilateral pheochromocytomas or multiple pheochromocytomas in the same gland. Paraganglioma arose in four (21.0%) patients. Two (10.5%) patients developed metastatic disease. PPGLs with *MAX* mutations were likely to generate predominantly excess norepinephrine [7]. In another article, PPGL with *MAX* mutation was characterized as a syndrome with high penetrance; the estimated penetrance reached to 73% by 40 years of age [8]. In our case, the patient was young without any family history of PPGL, and she had bilateral PPGL. As noted in previous report, her NM level was high, whereas metanephrine level was within normal range. These features are consistent with those described previously. Although various mutations have been identified in *MAX* so far [6, 7], the mutation of c.70_73 del AAAC/p.Lys24fs*40 has not been reported before. This mutation had led to a change in the predicted amino acid sequence; the lysine at codon 24 in the wild-type MAX protein was changed to a glycine in the mutant MAX protein, and a stop codon was located 40 codons downstream from codon 24. Thus, it is proposed that this newly detected mutation disrupts the MAX protein. In addition, in silico analysis using MutationTaster supported the pathogenicity of this deletion mutation.

Because aberrant MYC-MAX-MXD1 network is implicated in wide-range malignancies, it is worth to be noted that germline *MAX* mutation carriers may have increased susceptibility to develop other malignant tumors [9]. In the literature, two in 19 PPGL patients with *MAX* mutations had cancers, breast cancer, and squamous cell carcinoma of the tongue [7]. The patients of PPGL with *MAX* mutation need a close follow-up for not only recurrence of PPGL but also emergence of other malignant tumors. Recently, the crosstalk between PIK3CA/AKT1/mTOR and MYC/MAX/MXD1 pathways has been proposed [10]. The previous article speculates the effectiveness of mTOR blocking therapy for patients with malignant PPGL with *MAX* mutation [11].

The surgical indication of bilateral pheochromocytomas has remained controversial. In our case, we initially performed unilateral adrenalectomy to preserve her adrenocortical function in hope that unilateral adrenalectomy could ameliorate her symptoms. However, the symptoms recurred shortly after the operation. This decision was made based on the apparent unilateral accumulation in ^123^I-MIBG scintigraphy which indicated that the left pheochromocytoma was dominant. Although bilateral adrenalectomy was ultimately required to ameliorate her symptoms, we consider that our strategy, e.g., two-step adrenalectomy, was clinically acceptable. As a matter of course, a close follow-up after unilateral adrenal resection is essential.

The total adrenalectomy has been a standard treatment for unilateral or bilateral pheochromocytoma [12]. However, patients are put at risk of adrenocortical failure and need lifelong corticoid supplementation after total bilateral adrenalectomy. Recently, cortical sparing adrenalectomy in patients with *RET* or *VHL* mutations which had low risk of malignant PPGL has been reported [12, 13]. Accordingly, the recurrence rate after 10 years of follow-up was less than 5%, and a normal glucocorticoid function was retained in more than 50% cases [13]. In our case, because the left adrenal gland had multifocal pheochromocytomas and the medullas of both glands thickened, cortical sparing, in other words, partial adrenalectomy, seemed to be contraindicated due to high likelihood of metachronous occurrence of pheochromocytomas.

## Conclusions

We reported a rare case of PPGL with germline mutation in *MAX*. The surgical indication of bilateral pheochromocytomas should be decided considering each patient’s genetic background. A close follow-up for both recurrent PPGL and other types of malignant tumors is essential for the patients with *MAX* mutation.

## Abbreviations

CT: Computed tomography
MAX: MYC associated factor X
MIBG: Metaiodobenzylguanidine
NM: Normetanephrine
PPGL: Pheochromocytoma and paraganglioma
VMA: Vanillylmandelic acid

## References

[1] Welander J, Soderkvist P, Gimm O (2011). Genetics and clinical characteristics of hereditary pheochromocytomas and paragangliomas. Endocr Relat Cancer.

[2] Amar L, Bertherat J, Baudin E, Ajzenberg C, Bressac-de Paillerets B, Chabre O (2005). Genetic testing in pheochromocytoma or functional paraganglioma. J. Clin. Oncol. Off. J. Am. Soc. Clin. Oncol..

[3] Mannelli M, Castellano M, Schiavi F, Filetti S, Giacche M, Mori L (2009). Clinically guided genetic screening in a large cohort of Italian patients with pheochromocytomas and/or functional or nonfunctional paragangliomas. J Clin Endocrinol Metab.

[4] Atchley WR, Fitch WM (1995). Myc and max: molecular evolution of a family of proto-oncogene products and their dimerization partner. Proc Natl Acad Sci U S A.

[5] Grandori C, Cowley SM, James LP, Eisenman RN (2000). The Myc/Max/Mad network and the transcriptional control of cell behavior. Annu Rev Cell Dev Biol.

[6] Comino-Mendez I, Gracia-Aznarez FJ, Schiavi F, Landa I, Leandro-Garcia LJ, Leton R (2011). Exome sequencing identifies MAX mutations as a cause of hereditary pheochromocytoma. Nat Genet.

[7] Burnichon N, Cascon A, Schiavi F, Morales NP, Comino-Mendez I, Abermil N (2012). MAX mutations cause hereditary and sporadic pheochromocytoma and paraganglioma. Clin. Cancer Res..

[8] Bausch B, Schiavi F, Ni Y, Welander J, Patocs A, Ngeow J (2017). Clinical characterization of the Pheochromocytoma and paraganglioma susceptibility genes SDHA, TMEM127, MAX, and SDHAF2 for gene-informed prevention. JAMA oncology.

[9] Gimenez-Roqueplo AP, Dahia PL, Robledo M (2012). An update on the genetics of paraganglioma, pheochromocytoma, and associated hereditary syndromes. Horm. Metab. Res. = Hormon- und Stoffwechselforschung = Horm. Metab.

[10] Zhu J, Blenis J, Yuan J (2008). Activation of PI3K/Akt and MAPK pathways regulates Myc-mediated transcription by phosphorylating and promoting the degradation of Mad1. Proc Natl Acad Sci U S A.

[11] Cascon A, Robledo M (2012). MAX and MYC: a heritable breakup. Cancer Res.

[12] Iacobone M, Citton M, Viel G, Schiavone D, Torresan F (2017). Surgical approaches in hereditary endocrine tumors. Updat Surg.

[13] Castinetti F, Taieb D, Henry JF, Walz M, Guerin C, Brue T (2016). Management of endocrine disease: outcome of adrenal sparing surgery in heritable pheochromocytoma. Eur J Endocrinol.

